# Knowledge of physicians on chronic kidney disease and their attitudes towards referral, in two cities of Cameroon: a cross-sectional study

**DOI:** 10.1186/s13104-016-1845-5

**Published:** 2016-01-18

**Authors:** Simeon-Pierre Choukem, Pennghan K. Nchifor, Marie-Patrice Halle, Daniel N. Nebongo, Yannick Mboue-Djieka, François F. Kaze, Gotlieb L. Monekosso

**Affiliations:** Department of Internal Medicine and Pediatrics, Faculty of Health Sciences, University of Buea, Buea, Cameroon; Health and Human Development (2HD) Research Group, Douala, Cameroon; Diabetes and Endocrine Unit, Department of Internal Medicine, Douala General Hospital, P.O. Box 4856, Douala, Cameroon; Nephrology and Hemodialysis Unit, Department of Internal Medicine, Douala General Hospital, Douala, Cameroon; Department of Clinical Sciences, Faculty of Medicine and Pharmaceutical Sciences, University of Douala, Douala, Cameroon; Nephrology and Hemodialysis Unit, Yaoundé University Hospital Center, Yaoundé, Cameroon; Departement of Internal Medicine and Subspecialties, Faculty of Medicine and Biomedical Sciences, University of Yaoundé, Yaoundé, Cameroon

**Keywords:** Chronic kidney disease, Physicians, Knowledge, Attitude, Referral, Cameroon

## Abstract

**Background:**

Chronic kidney disease (CKD) is frequently unrecognized by non-nephrology physicians. There is an ongoing governmental program to create hemodialysis centers in the ten regions of Cameroon, where a previous study reported high levels of late referral to nephrologists. We aimed to assess the knowledge of physicians on CKD and their attitudes regarding referral.

**Methods:**

A questionnaire based on the Kidney Disease Outcome Quality Initiative Guidelines of 2002 was self-administered to general practitioners and non-nephrology specialists working in two Cameroon cities that have hemodialysis centers (Douala and Bamenda).

**Results:**

Of the 174 general practitioners and non-nephrology specialists approached, 114 (65.5 %) returned answered questionnaires. Only 58.8 % of doctors identified the correct definition of CKD. Most physicians were aware of the major risk factors of CKD (hypertension, 97.4 % and diabetes mellitus, 95.6 %). Most physicians were also aware of complications such as anemia (93.0 %), hypertension (90.4 %), uremia (85.1 %) and hyperkalemia (85.1 %). Only 44 % knew that CKD had five stages, with general practitioners 3.4 times more likely to know than specialists (p = 0.004). Even though 61.4 % of the physicians knew that the estimated glomerular filtration rate was the appropriate clinical means to diagnose CKD, 12.7 % would use serum creatinine alone for diagnosis. Also, up to 21.9 % of physicians would refer at late stage.

**Conclusion:**

General practitioners and non-nephrology specialists lack general knowledge on CKD, especially on the definition and staging; they also have inadequate attitudes with regards to diagnosis and referral to the nephrologists. Educational efforts are warranted to improve on physicians’ knowledge and skills on CKD in Cameroon.

**Electronic supplementary material:**

The online version of this article (doi:10.1186/s13104-016-1845-5) contains supplementary material, which is available to authorized users.

## Background

Chronic kidney disease (CKD) contributes significantly to the burden of diseases worldwide [1]. Due to its insidious onset and non-specific clinical manifestations, it often goes unrecognized and presents later on with severe complications that are multisystem. The prevalence of CKD worldwide has been estimated to be about 8–16 % [2]. In the United States of America, the prevalence of CKD in the adult population in 2003 was estimated at 11 % (19.2 million people) [3].

In sub-Saharan Africa (SSA), studies in Kinshasa in the Democratic Republic of Congo reported prevalence of 12.4 % in the general adult population [4] and up to 36 % in patients at high risk of CKD (hypertension, diabetes, obesity or HIV infection) [5]. However, reliable statistics on the prevalence of CKD in resource-limited settings like Africa are rare, with CKD still being an unrecognized health challenge [6]. Cameroon, like other SSA countries is facing a growing burden of hypertension and diabetes [7, 8], which are by far the major causes of CKD. It is therefore important to screening for CKD in high risk patients because early detection and management slows down the progression to end stage renal disease (ESRD). This can be achieved only if physicians are aware enough of the condition and its risk factors.

In the United States of America, primary care physicians were found to lack awareness on the kidney disease outcome quality initiative (KDOQI) guidelines, and were uncertain of what time was most appropriate for referral to a nephrologist [9]. A low level of awareness regarding time of referral has also been reported elsewhere (48.28 % in non-nephrology consultants and residents in Pakistan) [10].

Since 2008, Cameroon has developed a governmental program to build hemodialysis centers in the headquarters of the ten regions of the country. However, only 11 nephrologists are available to date, in a context of high rate (82.8 %) of late referral defined as estimated glomerular filtration rate (eGFR) <30 ml/min/1.73 m^2^ of patients with kidney impairment to nephrologists as reported in 2009 [11]. Late referral to nephrologists has been shown to cause 36 % excess death compared with early referral [12]. Assessing the level of knowledge and the attitudes of non-nephrology physicians is needed so as to inform the training and continuous medical education policy. The main objective of this study was to assess the knowledge of physicians on CKD and their attitudes regarding diagnosis and referral.

## Methods

### Study design and setting

This was a cross-sectional study carried out from March to July 2013 in two cities (Douala and Bamenda) that have hemodialysis centers. The city of Douala is the economic capital of Cameroon and is the most populous city of the country. It is home to the biggest hemodialysis unit of the country, created in 1990 and located at the Douala General Hospital. Bamenda is the headquarters of the North-West region of Cameroon and has a hemodialysis center since 2009. The total number of physicians in Cameroon was 1842 in 2011 [13]. At the time of the study, Bamenda had ten health facilities with a total of 78 physicians; the number of health facilities and physicians in Douala was estimated at 80 and 350 respectively. Physicians from all ten health facilities in Bamenda were invited to participate in this study. Physicians from Douala were invited to participate if they worked in the reference hospital (Douala General Hospital, total 70 physicians), the two most populous (in number of physicians, total 35 physicians) district hospitals, and four private health centers in the same two health districts (49 physicians).

### Data collection

The questionnaire used for this study consisted of 18 items (Additional file 1: Table S1), created based on the KDOQI guidelines of the National Kidney Foundation [14]. The questionnaire assessed knowledge on the definition, staging, risk factors and complications of CKD, markers of kidney function, forms of renal replacement therapy (RRT), laboratory investigations and timing of referral to a nephrologist. Questions were mainly closed-ended (a mixture of multiple choice and “Yes, No” questions) with just one open-ended question. Regarding the timing of referral to the nephrologist, referring at stages 1–3 was considered as “early referral” while referring at stage 5, referring when patients become symptomatic, or not knowing the time of referral was considered as “late referral”. Demographic data (gender, hospital of practice and level of training) were also obtained. The questionnaire was developed and reviewed by a team comprising an internist-nephrologist, an internist-endocrinologist and a statistician, and pilot-tested in ten physicians.

### Ethical approval

Ethical approval was obtained from the Institutional Review Board of the Faculty of Health Sciences, University of Buea, Cameroon. Administrative authorizations were issued by the Regional Delegations of Public Health of the Littoral (Douala) and the North-West (Bamenda) Regions. In each visited health facility,an additional authorization was also granted by the director before distribution of questionnaires. The questionnaire was anonymous with no demands for personal information. Physicians’ participation was voluntary, and only after they signed a written informed consent.

### Data analysis

Data were analyzed with STATA 11.0 (Statacorp, College Station, TX, 77845, USA). Results were presented as counts (percentages  %). Associations between variables were tested by the Chi square test or the Fisher’s exact test where appropriate. The significance threshold was set at a p value <0.05.

## Results

### General characteristics of study participants

Out of the 174 physicians approached (from a total of 232 who could be approached) 46 of 78 from the ten health facilities in Bamenda and 128 of 154 from seven health facilities in Douala, 114 (65.5 %) returned answered questionnaires 28 from the ten health facilities in Bamenda and 86 from the seven health facilities in Douala. Table 1 shows the main characteristics of participants. Among the 42 specialists, there were 15 from a discipline of internal medicine, 9 obstetricians/gynecologists, 9 surgeons, 5 pediatricians, 2 ear, nose and throat specialists, 1 anesthesiologist and 1 ophthalmologist.

**Table 1:** General characteristics of participants

Characteristics	Count	Percentage (%)
Gender (n = 114)		
Male	77	67.5
Female	37	32.5
Hospital of practice (n = 114)		
Public	80	70.2
Private	34	29.8
If private (n = 34)		
Confessional^a^	13	38.2
Lay private^a^	21	61.8
If public (n = 80)		
Medical centre	7	8.8
District hospital	19	23.8
Regional hospital	13	16.6
Reference hospital	41	51.3
Level of training (n = 114)		
General practitioners	72	63.2
Specialist	42	36.9

^a^Confessional hospitals are owned by religious organizations that place stringent control and emphasis on the way their patients are managed while the lay private hospitals are owned and managed with mostly a business focus. Confessional hospitals also send their doctors for refresher training whereas lay private often do not. Hence, doctors working in Confessional hospitals are generally expected to be better informed than their lay private colleagues

### Definition and staging of CKD

Out of the 114 physicians, 67 (58.8 %) selected the right definition of CKD. No association was found between the choice of the correct definition of CKD and gender of the physicians (p = 0.7), hospital of practice (p = 0.4), level of training (p = 0.5) or city of practice (p = 0.5).

One hundred and ten physicians responded to the question on staging of CKD, and 48 (44 %) selected the correct answer being 5 stages of CKD. Eleven physicians thought it had three stages, five thought it had four stages, one thought it had six stages and 45 did not know at all. General practitioners were 3.4 times more likely to give the correct answer compared to the specialists (Table 2).

**Table 2:** Factors associated with lack of knowledge on CKD stages

Factors	Wrong answer	Correct answer	p
Gender^a^			
Male	45 (59.2)	31 (40.8)	0.4
Female	17 (50.0)	17 (50.0)	
Hospital of practice^a^			
Public	45 (58.4)	32 (41.6)	0.5
Private	17 (51.5)	16 (48.5)	
Level of training^a^			
General practitioner	31 (45.6)	37 (54.4)	0.004
Specialist	31 (73.8)	11 (26.2)	

Data are presented as number (%)

^a^110 participants provided answers

### Risk factors and complications of CKD

Most physicians were aware that diabetes and hypertension were the major risk factors or causes, with 109 (95.6 %) and 111 (97.4 %) getting the right answers, respectively. A great proportion also identified glomerulonephritis (92.1 %) and drugs (90.4 %) as risk factors. HIV and hepatitis were less identified (57.0 and 30.7 % respectively).

Knowledge on the complications of CKD is shown in Table 3. The majority of respondents could identify most complications. Renal osteodystrophy was the least recognized while anemia was the most.

**Table 3:** Knowledge of complications of chronic kidney disease

Complications	Number of respondents	Percentage
Anemia	106	93.0
Hypertension	103	90.4
Hyperkalemia	97	85.1
Uremia	97	85.1
Coma	92	80.7
Edema	89	78.1
Nausea and vomiting	74	64.9
Osteodystrophy	63	55.3

### Forms of renal replacement therapy

Most physicians (96.5 and 93.0 %) were able to identify renal transplant and hemodialysis respectively as forms of RRT. Peritoneal dialysis was the least known form of RRT, with just 56.1 % aware of it.

### Diagnosis of CKD and timing of referral

In making a diagnosis of CKD, 12.7 % of the physicians chose to use the serum creatinine alone. Ten percent chose to use the serum creatinine and the eGFR. A greater percentage (25.5 %) chose to combine the serum creatinine, eGFR, a urinalysis and abdominal ultrasound scan to make the diagnosis of CKD. The rest of the physicians chose just a urinalysis, eGFR or abdominal ultrasound alone. Overall, only 61.4 % selected the eGFR alone or with other tests as the appropriate means of diagnosis.

Up to 21.9 % of the physicians chose to refer at a late stage or did not know when to refer (Table 4).

**Table 4:** Stage when physicians thought patients should be referred to a nephrologist

Stage of CKD	Number of respondents	Percentage
Stage 1	51	44.7
Stage 2	19	16.7
Stage 3	17	14.9
Stage 4	2	1.7
ESRD	1	0.9
Symptomatic	15	13.2
Need dialysis	2	1.7
I don’t know	7	6.1
Total	114	100

*CKD* chronic kidney disease, *ESRD* end stage renal disease

## Discussion

We have found in this study that less than three quarters (58.8 %) of the physicians interviewed were able to correctly define CKD. Less than half (44 %) knew that CKD has five stages, and this low rate was driven mostly by specialists (73.8 % of them gave wrong answers). We also observed that: more than 90 % of physicians were aware of the major risk factors of CKD, more than 80 % knew the main complications of CKD, and more than 90 % knew that hemodialysis and transplantation were means of RRT, whereas peritoneal dialysis that is not used in Cameroon was poorly known. As per the attitude, some physicians (12.7 %) still relied on serum creatinine alone for the diagnosis of CKD, and more than one-fifth (21.9 %) of physicians would still refer late.

In our context of limited number of nephrologists, most patients with CKD are seen by non-nephrologist physicians. Low CKD awareness implies that these patients are not properly managed to delay the progression to ESRD. Most of them will therefore very rapidly reach the stage of ESRD, thereby placing a huge burden on both the limited nephrologist workforce and the limited material resources available for RRT.

A proper understanding of the KDOQI definition of CKD is required to be able to make the diagnosis of CKD [14]. Rates reported in other developing countries are globally very low, varying from 38.8 % among non-nephrology specialists in Nigeria [15] to 48.8 in Pakistan [10]. Although the rate of 58.8 % found in our study is better, it still indicates a lack of awareness on the subject.

The KDOQI guidelines [14] classifies CKD into five stages based on the eGFR. Being able to identify them is vital for the development of an action plan for the patient, but less than half of our physicians (44 %) could do so. Studies in other developing countries show rates that vary from 42 % in Nigeria [15] to 73.6 % in Pakistan [10]. We also observed better responses from general practitioners as compared to specialists, probably because the formers studied medicine at the time the concept of staging of CKD was introduced. One way of raising the awareness of physicians on staging CKD is to recommend that laboratories should systematically calculate and even stage the eGFR on the serum creatinine results sheet. Such a strategy has proven to greatly increase the number of referrals to nephrologists [16, 17], but this is seldom done in Cameroon.

Diabetes and hypertension are well known as major risk factors of CKD, and emphasis is being laid on targeting these high risk groups for screening [18]. High proportions of the physicians correctly identified them as risk factors of CKD. Similar findings have been reported in Nigeria [15] and in American residents [19]. However, lower awareness regarding HIV and Hepatitis B and C virus infections which are also highly prevalent in SSA and highly involved in kidney diseases indicate a need to draw more attention towards their relationships with the kidney in medical training.

With a decline in kidney function and damage to renal parenchyma, the patient is exposed to complications such as anemia, hyperkalemia, edema, hypertension, osteodystrophy, gastrointestinal disturbances and eventually coma. Similar to findings by other authors [15, 20], anemia was the most commonly identified complication.

In SSA, hemodialysis is the most common form of RRT, although it is still underused [6]. Our respondents were highly aware of both renal transplant and hemodialysis as forms of RRT. However it is a matter of concern that despite the presence of hemodialysis in the study cities for many years, there were still physicians (7 %) who could not identify it as a form of RRT.

According to the KDOQI guidelines, the creatinine clearance should be calculated using eGFR equations [14]. More than one-tenth (12.7 %) of the physicians still chose serum creatinine alone for their diagnosis of CKD. This has potential negative clinical implications because older people may have altered kidney function with apparently normal plasma creatinine level.

Several studies have shown that late referral to the nephrologist is usually associated with adverse outcomes [11, 12, 21]. The reported rate of late referral defined as eGFR <30 ml/min/1.73 m^2^ in Cameroon is 82.8 %, irrespective of the source of referral [11]. In our study, more than 20 % of the physicians chose to refer patients late. The actual situation may even be worse because physicians had to choose one answer from a list, which could lead to a random selection of an appropriate answer. Using clinical scenarios could have provided close-to-real life results. Ideally, in a setting with very few nephrologists, it would be more cost-effective that patients at earlier stages of CKD benefit from nephrologist consultations and continue their follow up by primary care physicians who receive regular continuous medical education.

We acknowledge the following limitations: questions were limited because physicians were reluctant to provide some personal data despite explanations, and many open questions were excluded. Like any other knowledge and attitude study, ours was subjected to a self-reporting bias. However our study is a pioneer one that included more than 6 % of all Cameroon physicians; as such it adds new data on CKD in Cameroon, and results will certainly influence the training and continuous medical education policy with regard to kidney diseases. This continuing medical education of general practitioner and non-nephrologist specialists should lay emphasis on: primary (identify and control risk factors of CKD) and secondary (screen at-risk individuals, recognize CKD stages and manage it properly) prevention so as to allow them master when it is most appropriate to refer to a nephrologist either for a consultation, an advice, or for continuous follow up.

## Conclusions

General practitioners and non-nephrologists specialists have good knowledge regarding the main risk factors of CKD, its major complications and RRT. However, they are mostly unaware of the definition and staging of CKD. Also, they display poor attitude regarding diagnosis and timing of referral to the nephrologist. Our study suggests that training and continuous medical education should emphasize on the reported caveats. Also, systematic calculation and staging of eGFR by laboratories on serum creatinine result sheet could tremendously contribute in raising the awareness of physicians.

## Additional file

10.1186/s13104-016-1845-5 This table is the questionnaire used to collect data. It is divided into three sections. Section A (questions 1–3) focuses on identification: the type of hospital of practice, the gender and the level of medical training (general practitioner or specialist). Section B contains questions (4–9) on knowledge of chronic kidney disease (CKD). These questions cover the definition, the risk factors, the markers, the classification and the complications of CKD, and the types of renal replacement therapy. They are all multiple choices, with proposed answers to the participant who had to select which ever ones he/she thought were correct. Section C is made up of questions (10–18) on attitudes and practices. These questions cover the problem posed by CKD, the attitude regarding screening of patients at risk, the diagnosis of CKD and referral to the nephrologist. Questions are mostly multiple choices (questions 10–15, 17 and 18); one question (16) is opened.

## Abbreviations

CKD: chronic kidney diseases
eGFR: estimated glomerular filtration rate
ESRD: end stage renal disease
KDOQI: kidney disease outcome quality initiative
SSA: sub-Saharan Africa
RRT: renal replacement therapy
