# Supplementary material for: Knowledge of physicians on chronic kidney disease and their attitudes towards referral, in two cities of Cameroon: a cross-sectional study
Source: BMC Res Notes. 2016 Jan 18;9:29. doi: 10.1186/s13104-016-1845-5 (PMC4716638; doi:10.1186/s13104-016-1845-5)
Supplement: Supplementary file 1 — 10.1186/s13104-016-1845-5 This table is the questionnaire used to collect data. It is divided into three sections. Section A (questions 1–3) focuses on identification: the type of hospital of practice, the gender and the level of medical training (general practitioner or specialist). Section B contains questions (4–9) on knowledge of chronic kidney disease (CKD). These questions cover the definition, the risk factors, the markers, the classification and the complications of CKD, and the types of renal replacement therapy. They are all multiple choices, with proposed answers to the participant who had to select which ever ones he/she thought were correct. Section C is made up of questions (10–18) on attitudes and practices. These questions cover the problem posed by CKD, the attitude regarding screening of patients at risk, the diagnosis of CKD and referral to the nephrologist. Questions are mostly multiple choices (questions 10–15, 17 and 18); one question (16) is opened. [file 13104_2016_1845_MOESM1_ESM.docx]

**APPENDIX 1: QUESTIONNAIRE**

**QUESTIONNAIRE ON THE ASSESSMENT OF THE KNOWLEDGE, ATTITUDES OF PHYSICIANS WITH REGARDS TO CHRONIC KIDNEY DISEASE (CKD) IN DOUALA AND BAMENDA, CAMEROON**

**SECTION A: IDENTIFICATION**

**Date: /__/__//__/__//__/__/__/__/**

| 1 | Hospital of Practice | 1=Public, 2=Private |  |
| --- | --- | --- | --- |
|  | If private, specify if mission or not |  |  |
|  | If public, specify if sub-divisional, district, regional, reference |  |  |
| 2 | Gender | 0=Male, 1=Female |  |
| 3 | Level of Training | 1=General Practitioner, 2=Specialist |  |
|  | If specialist please specify what specialty |  |  |
|  | | |  |

**SECTION B: KNOWLEDGE**

| 4 | What is the definition of Chronic Kidney Disease (CKD) | 1= A condition of chronically elevated serum creatinine and urea which is usually reversible with appropriate management  2=Structural or functional kidney damage that can lead to impaired kidney function that persists for 3months or more with or without alteration of GFR  3=Irreversible and permanent elevation of serum creatinine  4= Elevation of serum urea  5= I don’t know  6=other (specify) |  |
| --- | --- | --- | --- |
| 5 | The following are risk factors of Chronic Renal failure (CRF) | Diabetes; 1=Yes, 2=No, 3-don’t know  Drugs; 1=Yes, 2=No, 3=don’t know  Hypertension; 1=Yes, 2=No, 3=don’t know  Glomerulonephritis; 1=Yes, 2=No, 3=don’t know  HIV; 1=Yes, 2=No, 3=don’t know |  |
|  |  | Hepatitis; 1=Yes, 2=No, 3=don’t know |  |
| 6 | The most appropriate marker for Kidney function is | 1=Serum Creatinine |  |
|  |  | 2= Creatinine clearance/Glomerular Filtration Rate |  |
|  |  | 3= Blood Urea Nitrogen |  |
|  |  | 4= Urine volume |  |
| 7 | The Kidney Disease Outcome Quality Initiative (KDOQI) Guidelines classify CKD into | 1= 1 stage  2= 2 stages  3= 3 stages  4= 4 stages  5= 5 stages  6= 6 stages  7= I don’t know |  |
| 8 | The following are complications of chronic renal failure | Anemia; 1=Yes, 2=No, 3=I don’t know |  |
|  |  | Hyperkalemia; 1=Yes, 2=No, 3=I don’t know |  |
|  |  | Uremia; 1=Yes, 2=No, 3=I don’t know |  |
|  |  | Hypertension; 1=Yes, 2=No, 3=I don’t know |  |
|  |  | Osteodystrophy; 1=Yes, 2=No, 3=I don’t know |  |
|  |  | Edema; 1=Yes, 2=No, 3=I don’t know |  |
|  |  | Nausea/vomiting; 1=yes, 2=No, 3=I don’t know |  |
|  |  | Coma; 1=Yes, 2=No, 3=I don’t know |  |
| 9 | The following are forms of Renal Replacement therapy (RRT) | Peritoneal Dialysis; 1=Yes, 2=No, 3=I don’t know |  |
|  |  | Kidney Transplant; 1=Yes, 2=No, 3=I don’t know |  |
|  |  | Hemodialysis; 1=Yes, 2=No, 3=I don’t know |  |

**SECTION C: ATTITUDES AND PRACTICES**

| 10 | Do you think CRF is a problem in Cameroon? | 1=Yes, 2=No, 3=I don’t know |  |
| --- | --- | --- | --- |
| 11 | If Yes, it is a | 1= Major problem |  |
|  |  | 2= Minor problem |  |
|  |  | 3= I don’t know |  |
| 12 | What is your attitude towards a patient at risk of developing CRF? | 1=Routine screening for CKD and management of risk factors.  2= Watchful waiting/No change in attitude |  |
| 13 | How often do you screen them? | 1= Every month  2= Every 6 months  3= Every year  4= Never |  |
| 14 | Kidney Disease is fatal | 1= Yes, 2=No, 3= I don’t know |  |
| 15 | What means do you use to make your diagnosis of CKD | Serum creatinine; 1=Yes, 2=No |  |
|  |  | Urinalysis; 1=Yes, 2=No |  |
|  |  | Glomerular Filtration Rate; 1=Yes, 2=No |  |
|  |  | Abdominal Ultrasound; 1=Yes, 2=No |  |
| 16 | If you have other means of diagnosing CKD, please specify |  | |
| 17 | When you diagnose CKD in a patient, what are your next steps | Patient education on the disease; 1=Yes, 2=No |  |
|  |  | Conservative management; 1=Yes, 2=No |  |
|  |  | Refer to a Nephrologist; 1=Yes, 2=No |  |
| 18 | In your opinion, at what stage should the patient be referred to a Nephrologist? | 1= Stage 1  2= Stage 2  3= Stage 3  4= Stage 4  5= At End Stage Renal Disease  6= When symptoms appear  7= When patient needs dialysis  8= I don’t know |  |

Please write overleaf in case you need more space
